# Intra-host evolutionary dynamics of the hepatitis C virus among people who inject drugs

**DOI:** 10.1038/s41598-021-88132-8

**Published:** 2021-05-11

**Authors:** Vincent Montoya, Anita Y. M. Howe, Weiyan Y. Dong, Winnie Dong, Chanson J. Brumme, Andrea D. Olmstead, Kanna Hayashi, P. Richard Harrigan, Jeffrey B. Joy

**Affiliations:** 1 British Columbia Centre for Excellence in HIV/AIDS, Vancouver, BC Canada; 2 British Columbia Centre for Disease Control, Vancouver, BC Canada; 3 Department of Medicine, University of British Columbia, Vancouver, BC Canada; 4 Faculty of Health Sciences, Simon Fraser University, Burnaby, BC Canada; 5 British Columbia Centre on Substance Use, Vancouver, BC Canada; 6 Bioinformatics Program, University of British Columbia, Vancouver, BC Canada

**Keywords:** Virology, Genome informatics

## Abstract

Most individuals chronically infected with hepatitis C virus (HCV) are asymptomatic during the initial stages of infection and therefore the precise timing of infection is often unknown. Retrospective estimation of infection duration would improve existing surveillance data and help guide treatment. While intra-host viral diversity quantifications such as Shannon entropy have previously been utilized for estimating duration of infection, these studies characterize the viral population from only a relatively short segment of the HCV genome. In this study intra-host diversities were examined across the HCV genome in order to identify the region most reflective of time and the degree to which these estimates are influenced by high-risk activities including those associated with HCV acquisition. Shannon diversities were calculated for all regions of HCV from 78 longitudinally sampled individuals with known seroconversion timeframes. While the region of the HCV genome most accurately reflecting time resided within the NS3 gene, the gene region with the highest capacity to differentiate acute from chronic infections was identified within the NS5b region. Multivariate models predicting duration of infection from viral diversity significantly improved upon incorporation of variables associated with recent public, unsupervised drug use. These results could assist the development of strategic population treatment guidelines for high-risk individuals infected with HCV and offer insights into variables associated with a likelihood of transmission.

## Introduction

Viral evolution is of critical clinical importance with multifaceted public health consequences including disease progression and virulence^1,2^, transmission dynamics^3,4^, and drug resistance^5,6^. Measures of viral population diversity can be measured within and among patients with the resulting metrics being informative for individual level treatment decisions and population level drug regimen choices^7,8^. Chronic RNA viral infections typically exist as a large population of diverse, inter-related viruses known as ‘quasispecies’^9^. For rapidly evolving RNA viruses such as the hepatitis C virus (HCV), it is estimated that each transcribed viral RNA molecule is unique relative to its progenitor due to its highly error prone RNA-dependent RNA polymerase^10,11^. While in general each mutation is randomly incorporated, signature patterns emerge as intense immune selective pressures placed upon each genome reflect specific stages of an HCV infection and determine the evolutionary fate of each mutation. Genomic diversity is relatively homogeneous during the early stages of infection as it has been shown that strong constraints on infectivity lead to a genetic bottleneck through selection of a single or limited number of founder variants^12,13^. Following the genetic bottleneck during initial infection, HCV genomic diversity increases rapidly over time as it adapts in response to novel host immune environs facilitating immune escape and viral persistence^14^. However, the increasing sequence diversity is counterbalanced by the need to maintain viral replication fitness.

Across the HCV genome, variation in intra-host diversity reflects both gene function and evolutionary rates between genes^9,15^. Genes encoding surface HCV antigens (e.g., envelope proteins E1 and E2) are under intense selective pressures from both humoral and cellular immunity which tend to have higher diversities, whereas the selective pressure on nonstructural protein regions, such as nonstructural protein 5B (NS5b), are predominantly cellular resulting in relatively lower diversities^16,17^.

Previous within-host studies of genetic diversity in HCV have largely focused on either clonal or deep amplicon sequencing targeting small portions of the HCV genome, such as the hypervariable region 1 (HVR) of the E2 gene or a 389 bp amplicon from the NS5b gene^12,14,15,18–21^. Of those employing a whole genome sequencing (WGS) approach, their primary research goals predominantly focus on phylogenetic or immunological associations^13,22–24^. Thus, research using WGS to investigate the relationship between viral diversity and duration of HCV infection among both chronic and acutely infected individuals is needed to compare the longitudinal evolution of each HCV gene region in order to determine which more accurately reflects time. Furthermore, how factors such as the transmission mode or how high-risk activities including recent injection drug use impact HCV diversification across the genome have not been adequately addressed. Accurate prediction of duration of infection based on HCV diversity could inform epidemiologically-derived estimates of HCV incidence and, in turn, help guide prevention, care, and treatment programming^25^.

In this study, we apply WGS retrospectively to a longitudinally sampled cohort from the Vancouver Injection Drug Users Study (VIDUS) cohort composed of individuals engaging in high-risk behaviours in order to identify regions of the HCV genome that correlate with duration of infection and determine the impacts of recent injection drug use upon these intra-host dynamics. We hypothesized that individuals engaging in high-risk activities such as recent injection drug use would display higher levels of intra-host HCV diversity due to higher propensities for re-infection, mixed infection, and higher variability among founder variants.

## Materials and methods

### Cohort description

The Vancouver Injection Drug Users Study (VIDUS) is a prospective open cohort that commenced enrollment in May 1996^26^. Subjects were recruited through street-based outreach and self-referrals within the downtown eastside of Vancouver (DTES), a region with a high prevalence of marginalized and vulnerable individuals, injection drug use, and HCV transmission^27^. Informed consent was obtained from all subjects involved in the study. Following provision of informed consent each subject with a history of injection drug use was invited to biannual study visits that involved an interviewer-administered questionnaire and blood draw for HIV and HCV antibody tests. These samples were linked with detailed socio-demographic and behavioural data derived from the questionnaires via a unique anonymous study identifier. Ethical approval for study of these samples, sequences, and socio-demographic data was obtained from the University of British Columbia Research Ethics Board (REB) specifically REB protocols H10-02004 and H11-03534. All methods performed in this analysis are in accordance with the relevant guidelines and regulations.

HCV samples were collected between 1996 and 2010 from participants in the VIDUS cohort who HCV seroconverted during the study. The date of HCV seroconversion, or duration of infection, was estimated using the midpoint of the first positive and last negative HCV antibody tests (time point 1). Longitudinal samples consisted of those collected at an average of 9 months after the estimated seroconversion (time point 2) as well as the final sample for each individual (time point 3). HCV viral loads were determined using an in-house qRT-PCR assay for all samples at the first time point^19^. In total there were 80 out of 106 individuals that yielded sufficient sequence data for analysis, while samples from 26 individuals failed to provide sufficient sequence data due to low viral loads, sample age, or clearance of the virus. Due to the unknown impacts of mixed infections upon viral diversity, all samples (n = 6) from two individuals with evidence of mixed infection were removed as well as single samples from four other individuals with genotype switches at the second or third time points. Of the remaining 124 samples from 78 individuals, 37 had sufficient sequence data at a single time point (median: 119 days post-infection, IQR: 92–119 days), two time points were available for 36 individuals (median: 416 days post-infection, IQR: 283–538 days), and three time points were available for five individuals (median: 1642 days post-infection, IQR: 1095–2500 days). Acutely infected individuals were those with an estimated duration of infection less than or equal to 6 months (184 days), whereas those estimated to be infected for greater than 6 months were classified as chronic infections. In total there were 28 acute samples and 96 chronic samples available for genomic analyses. Supplementary Figure 9 shows the distribution of the estimated durations of infections for all samples included in this study. While clinical and socio-demographic variables were available for all 78 individuals, variables such as mental illness, opioid treatment, history of alcohol abuse, and methadone treatment were not included in this analysis however since these variables were not available in the questionnaire across the entire sampling period.

### Nucleic acid extraction and sequencing

Near full length amplicons were generated using a modified protocol as previously described by Zhang et al.^28,29^. Briefly, HCV RNA previously stored at − 80 °C, was extracted using the NucliSENS easyMAG system (bioMérieux) according to the manufacturer's instructions. Reverse transcription was carried out in two steps. First, in order to remove secondary structures and facilitate sufficient binding of two reverse primers targeting the 3′ end of the HCV genome, extracted viral RNA was incubated at 65 °C for 5 min along with each primer. After cooling, an aliquot of this mix was then added to the master mix for cDNA synthesis using a standard Superscript III (Life Technologies) protocol. Following cDNA synthesis, two nested PCR reactions were carried out in order to generate near full-length amplicons. One nested PCR reaction generates an 8374 bp amplicon spanning the Core to the NS5B region and the other nested PCR reaction yields a partially overlapping 1193 bp amplicon at the extreme 3′ end of the HCV genome in order to span the remaining portion of the NS5B region. The PCR amplicons were then tagged and barcoded using Nextera XT index kits (Illumina, Inc.) and subsequently sequenced on an Illumina MiSeq (2 × 250 bp). However, diversities from the 8374 bp amplicon were only used in this study in order to minimize any bias between the two nested PCR assays.

### Variant calling

Consensus sequences were generated as described by Chui et al. using an automated bioinformatics pipeline, MiCall, freely available on Illumina’s BaseSpace^29,30^. In short, sequences are mapped using Bowtie2 to a set of 57 genotyped reference sequences that were phylogenetically selected in order to simultaneously include all HCV subtypes and minimize genetic overlap. A sample-specific consensus is generated and subsequently used as a reference in an iterative, adaptive mapping procedure that repeats until ≥ 95% of the reads are mapped or no additional reads are identified in a given mapping cycle. Thresholds for calling resistance-associated substitutions consisted of a depth of coverage of at least 100 reads with base quality scores ≥ 15 (Supplementary Table 1). This methodology has been shown to have high analytical sensitivities and high specificities for variant calling and consensus sequencing of viral genomes^30^.

### Calculating nucleotide diversity

Genomic diversity was measured using the Shannon entropy index at each nucleotide position of the genome with a minimum depth of 100 reads (Supplementary Equation 1). In order to identify the region of the genome with the highest correlation to the duration of infection, diversities for all combinations of consecutive positions of 100, 200, 300, 400, and 500 bp in length were used as well as the summation for each position within each singular gene, or in all possible combinations of genes in groups of two to nine (n = 1222 regions). Each gene region was also required to have > 99% coverage. Differences in the read depth between each position and/or sample were accounted for by normalizing the diversities by the log_10_ of the depth at each position. The median depth normalized Shannon diversities for each region were then used to assess correlations with the duration of infection as well as the status of each behavioural variable listed in Table 1. In order to account for differences in duration of infection for each individual's first and final time points, the depth normalized Shannon diversities were divided by the log_10_ of the duration of infection.

**Table 1:** Behavioural variables measured in this study for 78 participants.

Clinical variable	Individuals	Samples
HIV positive	9 (11.5)	10 (7.9)
Homelessness	29 (37.2)	36 (28.6)
Daily injection drug use in L6M	54 (69.2)	74 (58.7)
Injection drug use in L6M	71 (91)	106 (84.1)
Daily heroin usein L6M	35 (44.9)	49 (38.9)
Heroin in L6M	59 (75.6)	84 (66.7)
Daily cocaine use in L6M	22 (28.2)	25 (20)
Cocaine in L6M	52 (66.7)	70 (55.6)
Daily meth in L6M	4 (5.1)	4 (3.2)
Meth in L6M	14 (17.9)	16 (12.7)
Daily prescription opioid use in L6M	6 (7.7)	8 (6.3)
Prescription opioid use in L6M	25 (32.1)	32 (25.4)
Daily non-injection crack use in L6M	16 (20.5)	21 (16.7)
Non-injection crack use in L6M	36 (46.2)	53 (42.1)
Heavy alcohol use in L6M	5 (21.7)	6 (18.2)
Daily alcohol in L6M	10 (12.8)	11 (8.7)
Syringe sharing in L6M	25 (32.1)	31 (24.8)
Syringe borrowing in L6M	20 (25.6)	24 (19)
Syringe lending in L6M	18 (23.1)	22 (17.5)
Public drug use in L6M	50 (64.1)	66 (52.4)
Sex work in L6M	14 (17.9)	22 (17.5)
Any opioid use in L6M	8 (34.8)	9 (27.3)
Methadone treatment in L6M	27 (34.6)	34 (27)
Jail in L6M	26 (33.8)	32 (25.6)
History of mental illness	27 (44.3)	37 (42)
Mental illness in L6M	7 (12.1)	7 (8.2)
Daily marijuana use in L6M	18 (23.1)	24 (19)
Marijuana use in L6M	48 (61.5)	78 (61.9)
Living in the downtown eastside	39 (50)	58 (46)
Any resistance mutation*	26 (33.3)	42 (33.3)
Q80K resistance*	21 (26.9)	34 (27)

Counts for all behavioural variables available for analysis in this study for each individual/sample as well as the percent positive (in parentheses).

*Measured from deep sequencing analyses. L6M represents last 6 months prior to sample collection. Positives for each variable are defined as positive at any point during sampling.

### Statistical analyses

All statistical analyses were performed in R version 3.5.1^31^. Mann–Whitney–Wilcoxon tests were used to compare each behavioural variable with each diversity measure for the first and last samples from each subject. Selection of minimally adequate models based on the Akaike information criterion (AIC) for each behavioural variable was performed using both a forward and reverse stepwise algorithm (stepAIC function) from the MASS package^32^. Pearson correlation tests were used to assess correlations among behavioural variables in R. Intra-host diversification rates were calculated using the first and last samples for each individual, where the change in the Shannon diversities was divided by the natural log-transformed change in duration of infection. Receiver operating characteristic curve analyses were performed with the pROC package in R^33^.

### Phylogenetic analyses

**Figure 1:**
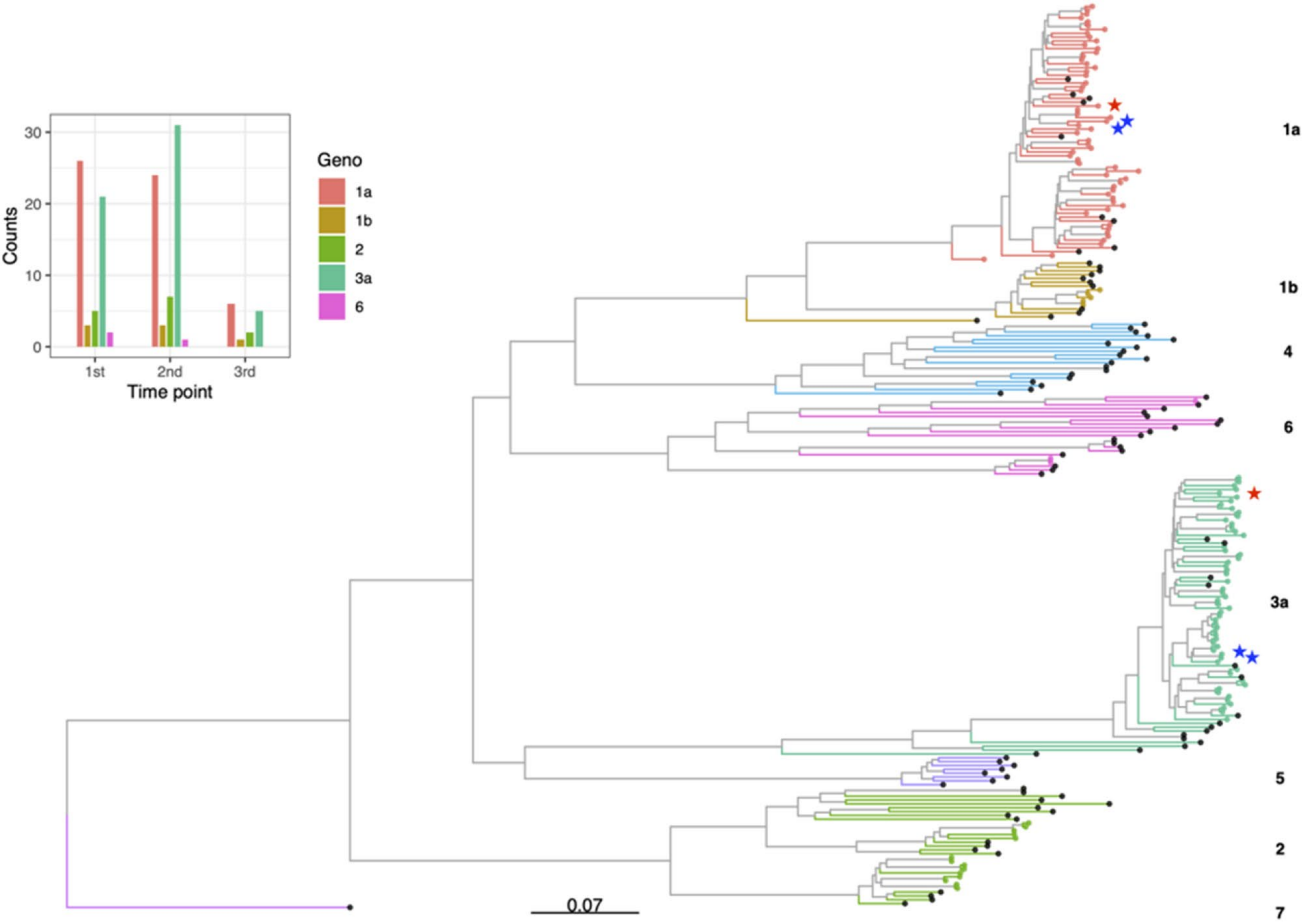
Phylogenetic tree of HCV consensus sequences and genotype counts. Maximum likelihood phylogenetic tree of HCV WGS. Reference genomes for each genotype are shown with black points and different coloured tips signify those included in this study. This tree was rooted on a genotype 7 reference sequence. Stars indicate mixed infections which were removed from subsequent analyses, red for sample 33 and blue for sample 983 (two longitudinal samples). Inset bar chart depicts counts of genotypes for each sample (including mixed and genotype switches) separated by time point.

Multiple sequence alignment was performed using MAFFT v7.54^34^. The resulting alignment was visually inspected and checked for errors using AliView 1.26^35^. Subsequently, an approximate maximum likelihood phylogenetic tree was inferred under a general time reversible model of molecular evolution from the consensus sequences using FastTree version 2.1.10. The resulting tree was rooted on an HCV genotype 7 reference sequence (Genbank accession NC_030791) and plotted using the ggtree package (Fig. 1)^36,37^.

## Results

### General features of near full-length HCV amplicons

Of the 78 participants with sequence data available for whole genome analyses, 43 were sampled longitudinally at a maximum of three time points ranging from 30 to 2500 days apart. The average depth ranged from 232 to 10,400 reads (median = 4515). Although four genotypes were identified (1, 2, 3, and 6), most samples were subtype 1a (40.8%) or subtype 3a (41.6%) (Fig. 1). In total there were two potential mixed infections, where both individuals were infected simultaneously with subtypes 1a and 3a (Supplementary Table 2, Supplementary Figure 3). In three other individuals, the genotypes switched over time (1a > 3a in 2 subjects, and 6e > 1a in 1 subject). Despite being part of a treatment naïve cohort, there were 27 individuals with known resistance-associated sites (RAS), predominantly Q80K and M175L within the NS3 gene (Supplementary Figure 4), and a low level (< 5%) of RAS within the NS5a gene. RAS have previously been observed in the absence of drug pressures for HCV infected individuals^38^.

### Genomic diversity, behavioural variables, and duration of infection

**Figure 2:**
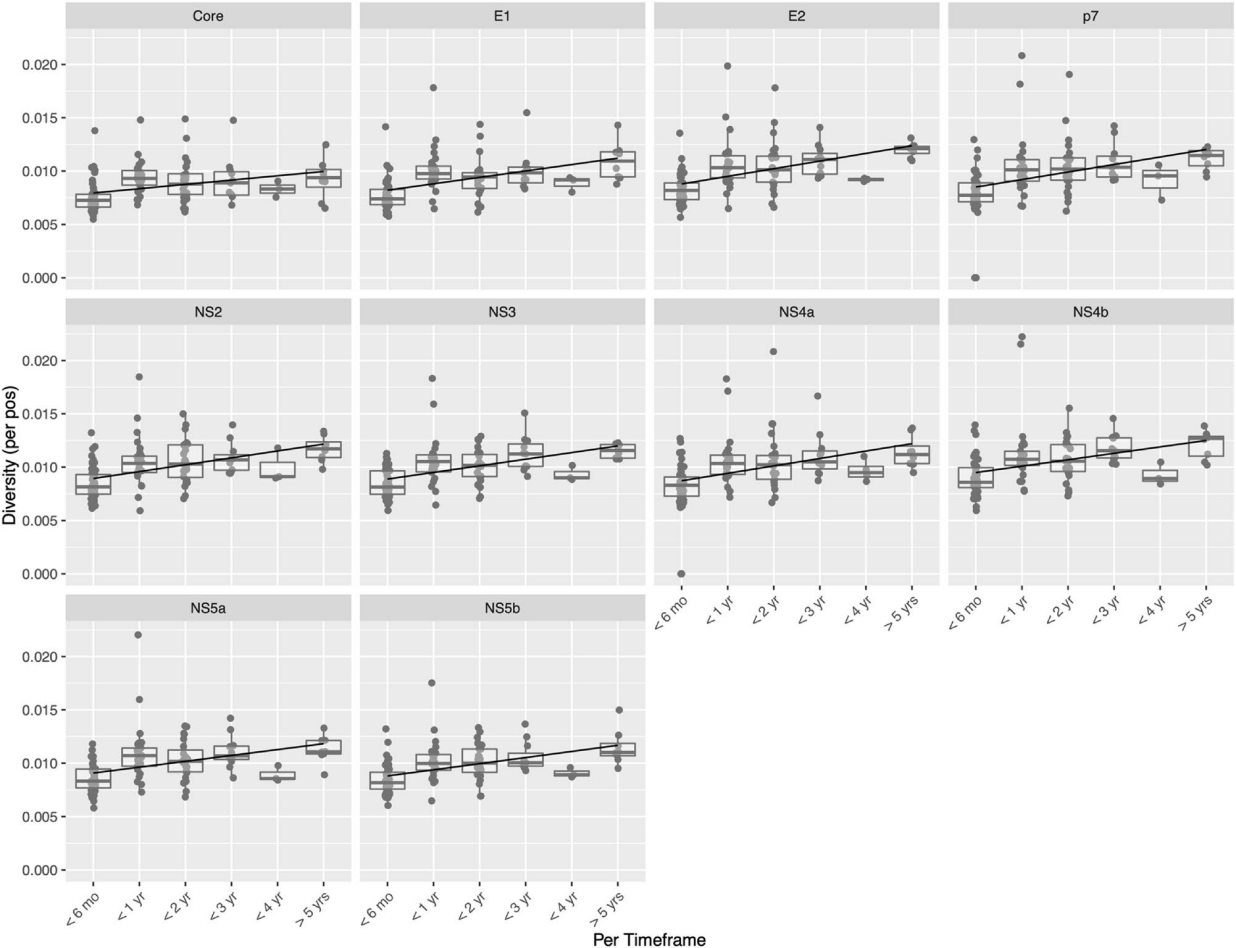
Shannon diversity across the HCV genome and over time. Read depth normalized Shannon diversities for each gene grouped by timeframe of infection: less than 6 months (< 6 mo), between 6 months and 1 year (< 1 yr), between 1 and 2 years (< 2 yr), between 1 and 3 years (< 3 yr), between 3 and 4 years (< 4 yr), and greater than 5 years (> 5 yrs).

Shannon diversities at each nucleotide position were calculated for each sample (Supplementary Figure 5). Viral diversity generally increased over the three time points (Supplementary Figure 8) and over time (Fig. 2). The calculated viral diversities for each sample showed little correlation with viral loads (Supplementary Figure 1).

Previous studies correlating duration of infection with intra-host genomic diversity have focused predominantly on short segments of the HCV genome. For this WGS analysis, a sliding window approach was implemented where diversities were averaged over 100, 200, 300, 400, and 500 bp regions in addition to average of diversities for both each singular gene and in all combinations of genes. Linear models were applied to all gene regions to test if diversity could predict duration of infection. Results from a linear mixed model analysis accounting for within-host correlations displayed no significant differences suggesting intra-host correlations were not significantly influencing this relationship (Table 2, Supplementary methods). Similarly, when examining one sample from each individual (preferentially selecting the earliest acute infections and the final chronic samples, henceforth referred to as the single sample acute group), the resulting model fits were similar. Among each window across the genomes in this study, the NS3 region was identified as the most clock-like region with an AIC ranging from 279.9 to 285.05 (Table 2, Supplementary Figure 12).

**Table 2:** Akaike information criterions for models predicting time from diversity.

Gene	Window	lm AIC	MM AIC	lm R^2^
NS3	499:598	279.90	281.66	0.27
NS3	500:599	279.67	281.44	0.27
NS3	1561:1660	285.05	286.49	0.23
E1	263:462	284.81	284.33	0.24
NS3	526:825	286.25	287.87	0.23
NS5a	817:1116	291.80	292.96	0.19
NS5b	702:901	290.16	292.16	0.20
NS5a	750:1149	292.74	293.78	0.18
NS3	1205:1604	291.12	292.56	0.19
NS3	1215:1414	291.98	293.52	0.19
NS5b	657:756	292.84	294.46	0.18
E2	1:81	301.41	303.34	0.12

Shown are the linear mixed model AICs (MM AIC), the linear model AIC (lm AIC), and the linear model R^2^ values (lm R^2^). The models with the best AICs are shown among each region. There were additional overlapping regions within each 100, 200, 300, 400, and 500 bp window containing similar AIC values, however unique regions are preferentially displayed.

### Influence of behavioural variables and genomic diversity

It is conceivable that high-risk behaviours such as those associated with habitual intravenous drug injection could lead to a more diverse intra-host viral population and therefore distort investigations of diversity and time. In order to test this hypothesis prior to exploring the relationship of diversity and time further, the relationships between the diversities among those engaging and not engaging in each of the behavioural variables listed in Table 1 were examined for the first and final time points of each individual. Diversities were significantly elevated among individuals engaging in recent heroin/injection drug/cocaine use only after adjusting for duration of infection (Table 3, see “Materials and methods” section, Supplementary Figures 6–7). Interestingly, those individuals engaging in recent heroin use displayed elevated diversities at both their first and final time points, whereas those engaging in recent injection drug and cocaine use displayed elevated diversities in their final time points. These differences suggest that the rate of diversification (the changes in intra-host viral diversification over the change in time) might also be significantly different, however no significant differences were identified.

**Table 3:** Significant changes in viral diversity due to behavioural variables.

Gene	Variable	1st sample	Last sample
Core	Heroin in L6M	**0.02**	**0.02**
E1	Heroin in L6M	**0.02**	**0.03**
NS3	Heroin in L6M	**0.01**	**0.04**
NS4a	Heroin in L6M	**0.03**	0.07
NS4b	Heroin in L6M	**0.01**	0.07
NS5a	Heroin in L6M	**0.03**	0.06
NS5b	Heroin in L6M	**0.03**	**< 0.05**
Core	Injection drug use in L6M	**0.03**	**< 0.01**
NS3	Injection drug use in L6M	0.17	**0.04**
NS5b	Injection drug use in L6M	0.19	**0.03**
Core	Cocaine in L6M	0.17	**< 0.01**
E1	Cocaine in L6M	0.28	**0.04**
NS4b	Cocaine in L6M	0.27	**< 0.05**
NS5b	Cocaine in L6M	0.37	**0.03**
Core	Daily cocaine in L6M	0.13	**< 0.01**
NS4b	Daily cocaine in L6M	0.32	**0.03**
NS5b	Daily cocaine in L6M	0.5	**0.03**

Diversities were compared for each individual's first and final time points for each group using a Mann–Whitney–Wilcoxon test. L6M—last 6 months. Significance was determined with *p* values less than 0.05 (in bold).

### Models predicting duration of infection from viral diversity are improved upon the incorporation of recent drug use status

**Figure 3:**
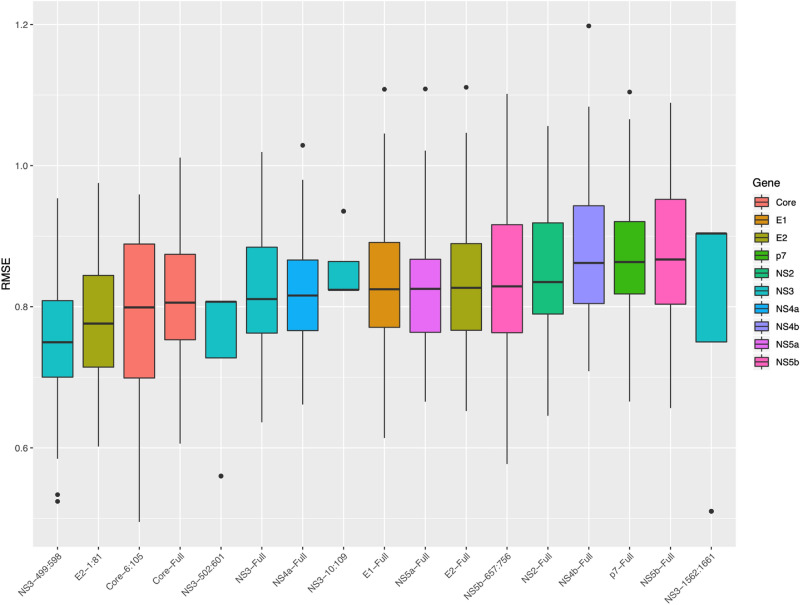
Measuring the time to infection prediction capacity of diversities from each gene/region. Shown are the root mean square error (RMSE) values used to measure the capacity of diversities from each region to predict duration of infection. The top 17 models determined by their median RMSE values are shown for all regions tested.

Collectively, these limited differences in diversities among recent injection drug users support the hypothesis that high-risk behavioral variables associated with transmission should be accounted for when modelling the relationship between viral diversification and duration of infection. Thus, all behavioural variables were included in each of the linear models in an iterative fashion until the best performing model was identified (see “Materials and methods” section). Despite the significance of heroin use described above, only public drug use, homelessness, and cocaine use were found to improve model fits (Table 4). Similar to previous models, the NS3 gene was identified as the region with the highest correlation to time. Furthermore, the same NS3 region was shown to be the most predictive of duration of infection when the data was split into a training (75%) and test (25%) data set (Fig. 3, Supplementary methods).

**Table 4:** Linear models with and without behavioural variables.

Gene	Window	Raw AIC	Step AIC	Step AIC R^2^	Variables
NS3	499:598	279.90	259.17	0.46	Public drug use
NS3	500:599	279.67	259.22	0.45	Public drug use
NS3	1561:1660	285.05	260.94	0.45	Cocaine + public drug use + any res
E1	263:462	284.81	265.83	0.42	Homelessness
NS3	526:825	286.25	266.09	0.42	Public drug use
NS5a	817:1116	291.80	272.12	0.39	Homelessness + public drug use
NS5b	702:901	290.16	272.41	0.38	Homelessness + public drug use
NS5a	750:1149	292.74	272.57	0.39	Public drug use
NS3	1205:1604	291.12	273.05	0.39	Homelessness + public drug use
NS3	1215:1414	291.98	274.07	0.37	Homelessness + public drug use
NS5b	657:756	292.84	274.48	0.37	Homelessness
NS5b	658:757	292.99	275.00	0.37	Homelessness
E2	1:81	301.41	284.15	0.31	Homelessness

Models were built for each full gene as well as for each specified region. AICs are shown for linear models without clinical data generated from the data set (Raw AIC) as well as when all variables were included (step AIC) along with the R^2^ values for the step AIC models (stepAIC R^2^). Any Res. are samples with any resistant positions detected.

### Identification of acutely infected individuals

The ability of each window across the HCV genome to differentiate acute from chronic infections was also evaluated using the area under the receiver operating characteristic curve (AUC). Interestingly the highest AUC value (0.85) was identified in the NS5b region (657–756, H77 coordinates: 8258–8357), closely followed by a region in the NS3 gene (1215–1414, H77 coordinates: 4634–4833) (Table 5, Supplementary Figure 13). The previously identified NS3 region with the most clock-like relationship with diversity had a lower AUC of 0.80. These results also reflect a similar analysis performed using only single samples from each individual (see “Materials and methods” section) suggesting that the results were not significantly influenced by within-host relationships.

**Table 5:** Area under the receive operating characteristic curves for the differentiation of acute from chronic infections.

Gene	Window	Total samples	Single sample acute group
NS5b	657:756	0.85	0.85
NS3	1215:1414	0.84	0.85
NS5b	702:901	0.84	0.84
NS5a	750:1149	0.84	0.83
NS3	470:969	0.81	0.81
NS3	499:598	0.80	0.81
NS3	1561:1660	0.80	0.81
E2	1:81	0.79	0.81
E1	263:462	0.78	0.79

Total samples are those AUC values obtained from analyses using the total samples from this dataset, whereas 'single sample acute group' removes longitudinal data and retains the earliest acute and latest chronic samples of each individual.

## Discussion

In this study, 124 genomes were deep sequenced from 78 individuals with a history of injection drug use and the diversities for all gene regions were analyzed over three different time points. While diversities from many regions were generally correlated to duration of infection, the NS3 protease was consistently most ‘clock-like’ across all models. However, concerning the ability to differentiate acute from chronic infections, a 100 bp region within the NS5b gene was most efficient. Viral populations within individuals engaging in recent injection drug use were shown to have higher diversities relative to their respective control groups. Upon the incorporation of recent public drug use (unsupervised drug use) and/or homelessness status into models testing whether diversity can predict duration of infection, there was a significant improvement in the overall model fit.

While the immune system is a strong driver of viral diversification, these processes are not linear with respect to time. Antibodies specific to HCV are detectable during acute infection however their selective pressures are thought to be exerted predominantly during the chronic phase of infection whereas the selective pressures of T cell responses are thought to occur during earlier stages of infection^39,40^. During the chronic phase of the infection a waning T cell response has been observed and thought to occur due to immune exhaustion^41,42^.

The limited humoral response directed upon the nonstructural antigens regions highlighted in this study could imply that antibody-dependent processes such as affinity maturation and somatic hypermutation that occur weeks or years after the acute phase of infection^43,44^, may place significant selective pressures upon surface antigens that can ultimately lead to variable changes in the general population structure in a temporal manner. Further research is needed to address how these specific selective pressures impact viral diversities of both structural and nonstructural antigens over the course of infection and how these are counterbalanced by the requirements of the virus to maintain replication fitness.

Each of the NS3 and NS5b regions highlighted in this study include critical domains within each of their respective encoded proteins and are known to be targeted by the hosts T cell immune response. The NS3 region producing diversities with the highest correlation to time (H77 coordinates: 4634–4733) encompasses both the C-terminus of the protease domain and the N-terminus of the helicase domain^45^ as well as an overlapping CD4+ T cell antigen^46^. The NS5b region in question (H77 coordinates: 8258–8502) contains several interesting features including (a) motifs within the active site of the RNA polymerase (b) one of the primary sites of selection in the presence of the antiviral drug sofosbuvir, S282, and (c) a CD4+ T cell antigen reported to be targeted by those with resolved infections^47–49^. While these regions were shown to outperform other regions in terms of correlations to time, there were several other regions exhibiting competitive model fit values as well as differentiation capacity as measured with receiver operating characteristic curves. Nevertheless, it would be interesting to investigate in future studies why these particular regions show higher correlations with duration of infection when compared with other regions that have similar requirements to HCV replication and host immune selective pressures.

The NS5b region highlighted in this study has previously been used to differentiate acute from chronic infections in HCV^50,51^. In these previous studies, a 389 bp amplicon was used for incidence estimations whereas in this study an 8374 bp region was amplified. While PCR amplifying a relatively small amplicon can significantly increase sequencing depths relative to larger genomes and/or amplicons, the evolution of the remaining portion of the genome is ignored. Nevertheless, the results from this study suggest that if the goal of a particular research project is to identify recent infections then this 389 bp amplicon is a worthy candidate. A recent publication using the HVR of the HCV genome to identify recent infections (in this case < 1 year since infection) impressively reported accuracies > 95%^20^. These results suggest that using a combination of metrics, including the physical chemical features of a nucleotide sequence, may enhance both sensitivity and specificity for incidence estimations. However, in Baykal et al., it was unclear what the durations of infections as well as the range of sequencing depths were for each of the samples and/or groups in question. While the study population was sufficiently large enough (98 recently/256 persistently infected) such that these measures should not significantly influence the results, it is worth noting that each of these measures can have significant impacts on subsequent analyses and therefore must be clearly stated and accounted for in future studies with similar research aims. In the present study, nucleotide diversities from ~ 93% of the protein encoding region of the HCV genome were examined for their differentiating potential and the HVR region was found to be outperformed by multiple alternative regions. Interestingly however, while there were no significant differences among all of the top-performing regions (including the HVR) when specifically testing their predictive capacity, the HVR predicted time to infection with the second lowest median root mean squared error. Ultimately, an analysis with a side-by-side comparison of each region using similar analytical metrics is likely required.

It has been demonstrated that a genetic bottleneck occurs during HCV transmission where a single or a limited number of founder variants are transmitted^13^. Similar to HIV^52^, we hypothesized that for individuals engaging in injection drug use, a larger population of HCV virions would be transmitted at a higher frequency relative to other modes of transmission and thus would correspond to higher measures of genetic diversity in the recipient. Opiates in general are thought to have immunosuppressive effects both indirectly through the central nervous system and directly through inhibitory actions of cellular and antibody immune responses^53^, and therefore changes to viral population structures would not be unexpected. However, the extent to which each individual’s viral population diversifies specifically due to injection drug use, the downstream effects of this activity, or simply the background of each individual hosts immunological status is unknown. Upon comparing the first and final samples from each individual in this study, recent heroin, injection drug, and cocaine use all showed elevated diversities compared to each of their respective control groups. However, only recent public drug use (unsupervised drug use) as well as homelessness were shown to significantly improve models predicting duration of infection from viral diversities for several regions of the HCV genome. While the inconsistency in these results limit the conclusions from this study, they collectively suggest that certain high-risk behaviours may have significant changes to intra-host viral diversities in a temporal fashion.

Due to the paucity of sampling of this injection drug user cohort in Vancouver, the observed differences in each of the behavioural variables in this exploratory study need to be examined further using a larger sample size with sufficient statistical power. Importantly, a limitation of this study is that the control group for the behavioural variables accounted only for the 6 months prior to sampling. Since injection drug use was a requirement for recruitment in this study, all individuals who did not inject drugs during this period were previously injecting drugs. Previous research has found that drug use cessation among VIDUS participants has significantly increased over the duration of the study and coincides with increases in the availability of needle and syringe exchange programs^54^. Further studies are needed to assess the evolution of intra-host viral populations between those with and without a history of injection drug use to properly address this question.

In conclusion, this study demonstrates that the intra-host evolutionary dynamics of HCV in the absence of direct acting antivirals behave generally in a clock-like fashion even among those engaging and re-engaging in high-risk activities. Although current direct-acting antiviral regimens provide high efficacy rates to most patients including injection drug users, it is unlikely that this “sustained virologic response (SVR)” offers long-term immunity. Many studies have shown that HCV reinfection can occur after SVR among people who inject drugs suggesting that there is limited or no protective immunity subsequent to therapeutic cure. A complete elimination and prevention of HCV infection strategy may require an effective vaccine. Our results reveal that genetic diversity and intra-host viral evolution can be different for recent injection drug users. The immunological pathways orchestrating these viral-host interactions is unknown, but it is conceivable that an effective vaccine would need to take these differences in viral diversity into account. Understanding the host determinants driving host immunity against HCV infection will be essential in the design of an optimal vaccine strategy.

## Supplementary Information

Supplementary Information.
