# Supplementary material for: Intra-host evolutionary dynamics of the hepatitis C virus among people who inject drugs
Source: Sci Rep. 2021 May 11;11:9986. doi: 10.1038/s41598-021-88132-8 (PMC8113533; doi:10.1038/s41598-021-88132-8)
Supplement: Supplementary file 1 — Supplementary Information. [file 41598_2021_88132_MOESM1_ESM.docx]

**Supplementary Material**

**Intra-host evolutionary dynamics of the hepatitis C virus among people who inject drugs**

Vincent Montoya^1^, Anita YM Howe^2^, Weiyan Dong^1^, Winnie Dong^1^, Chanson J. Brumme^1,3^, Andrea D. Olmstead^2,3^, Kanna Hayashi^4,5^, P. Richard Harrigan^3^, Jeffrey B. Joy^1,3,6*^

^1^British Columbia Centre for Excellence in HIV/AIDS, Vancouver, BC, Canada

^2^British Columbia Centre for Disease Control, Vancouver, BC, Canada

^3^Department of Medicine, University of British Columbia, Vancouver, BC, Canada

^4^Faculty of Health Sciences, Simon Fraser University, Burnaby, BC, Canada

^5^British Columbia Centre on Substance Use, Vancouver, BC, Canada

^6^Bioinformatics Program, University of British Columbia, Vancouver, BC, Canada

*Corresponding author: jjoy@bccfe.ca

**Supplementary Methods**

*Comparing Linear mixed models with standard linear model*

In order to identify the highest correlation to time among the diversities for each region as well as each full-length gene, linear mixed models were initially used to account for autocorrelations between each longitudinal sample. However, with a data set composed of a maximum of three longitudinal samples, it can be difficult to accurately estimate the true variance for a given individual. Linear mixed models were simplified in a step-wise manner using the Akaike Information Criterion (AIC) where the variables duration of infection, individual, and genotype were used as fixed and random effects. Prior to running the models, the distribution of duration of infections was examined for each data set. In order to reduce the skewness, duration of infection was natural log-transformed which also ensured that residuals were homogenous and normally distributed. For both linear mixed and traditional linear models, normality and error variances were assessed by the Shapiro-Wilk test as well as through visual inspection of residuals plotted against predicted values. A random intercept linear mixed model was selected to test if diversity could predict duration of infection for each genomic region, where each individual was set as the random effect to account for repeated measures. All linear mixed models were constructed using the ‘lmer’ functions in the lme4 package version 1.1-21 and the linear models were generated with the lm function in R [32].

In order to ascertain the potentially beneficial changes to each mixed model, variables from the stepwise algorithm used with the linear models (see main text).

*Predicting duration of infection from viral diversity*

All 100 bp regions and full genes (individually and all combinations) were analyzed further to assess their ability to predict duration of infection. In order to evaluate the capacity of each region, each respective data set was split into a training data set (75%) to build the models and a testing data set (25%) for prediction assessment. The variables previously identified in the full data set that were found to improve models most significantly were used for both the training and test data sets. The accuracy of the linear model using the training data set to predict the duration of infections in the test data set was assessed using the root mean square error. Among the most predictive genes/regions, the top seven were found within the same NS3 regions previously identified in the linear model analysis (only one region is shown in main Figure 3, main Table 4).

**Supplementary Tables and Figures**

| Gene | Pos | 1a | 1b | 2 | 3 | 4 | 5 | 6 |
| --- | --- | --- | --- | --- | --- | --- | --- | --- |
| NS5b | 159 | L159F | L159F | L159F | L159F |  |  |  |
| NS5b | 282 | S282T/R | S282T | S282T | S282T | S282T | S282T |  |
| NS5b | 320 | L320I/F/V |  |  |  |  |  |  |
| NS5b | 321 | V321A |  |  | V321A |  |  |  |
| NS5a | 24 | K24G/N/R |  | T24A |  |  |  | Q24H |
| NS5a | 26 | K26E |  |  |  |  |  |  |
| NS5a | 28 | M28A/G/T/S/V | L28M/T | L/F28M/V/S | M28T | L28S/V | L28I | F28L |
| NS5a | 29 |  | P29S |  |  |  |  |  |
| NS5a | 30 | Q30C/D/E/G/H/I/K/L/Q/R/S/T/Y | R30G/H/P/Q/R | L30H/S | A30K/S | L30H |  |  |
| NS5a | 31 | L31I/F/M/P/V | L31F/I/M/V | L31M/V | L31I/M/V | L31I/M | L31V | L31M/V |
| NS5a | 32 | P32L/S | P32F/L/S |  |  |  |  | P32L/S |
| NS5a | 38 | S38F |  |  |  |  |  |  |
| NS5a | 58 | H58D/L/R | P58D/S |  |  | T58P/S |  | T58A/N/S |
| NS5a | 62 |  | Q/E62D |  |  |  |  |  |
| NS5a | 92 | A92K/T | A92K |  |  |  |  |  |
| NS5a | 93 | Y93C/F/H/L/N/R/S/T/W | Y93C/H/N/S | Y93H | Y93H | Y93H/R |  | V36I |
| NS3 | 36 | V36A/C/G/L/M | V36A/C/G/L/M |  |  |  |  |  |
| NS3 | 41 | Q41R | Q41R |  |  |  |  |  |
| NS3 | 43 | F43L | F43I/S/V |  |  |  |  |  |
| NS3 | 54 | T54A/S | T54A/C/G/S |  |  |  |  |  |
| NS3 | 55 | V55A/I | V55A |  |  |  |  |  |
| NS3 | 56 | Y56H | Y56H/L | Y56H |  | Y56H |  | Y56H |
| NS3 | 80 | Q80H/K/L/R | Q80H/K/L/R |  |  |  |  | L80K/Q |
| NS3 | 122 | S122G/R | S122D/G/I/N/R/T |  |  |  |  | S122T |
| NS3 | 155 | R155G/I/K/M/S/T/W | R155C/G/I/K/Q/M/S/T/W |  |  |  |  |  |
| NS3 | 156 | A156S/T/V | A156G/F/S/T/V |  |  |  |  |  |
| NS3 | 158 | V158I | V158I |  |  |  |  |  |
| NS3 | 168 | D168A/C/E/F/G/H/I/K/L/N/T/V/Y | D168A/C/E/F/G/H/I/K/L/N/T/V/Y |  | Q168R | D168V |  | D168E/Y |
| NS5b | 170 | V170F/I/T/V | V170A/I/L/T |  |  |  |  | I170V |
| NS5b | 175 |  | M175L |  |  |  |  |  |
| NS5b | 314 | L314H |  |  |  |  |  |  |
| NS5b | 316 | C316Y | C316H/N/Y/W |  |  |  |  |  |
| NS5b | 368 |  | S368T |  |  |  |  |  |
| NS5b | 411 |  | N411S |  |  |  |  |  |
| NS5b | 414 | M414I/T/V | M414I/T/V |  |  |  |  |  |
| NS5b | 445 |  | C445F/Y |  |  |  |  |  |
| NS5b | 446 | E446K/Q |  |  |  |  |  |  |
| NS5b | 448 | Y448C/H | Y448C/H |  |  |  |  |  |
| NS5b | 451 | C451R |  |  |  |  |  |  |
| NS5b | 553 | A553T | A553V |  |  |  |  |  |
| NS5b | 554 | G554S | G554S |  |  |  |  |  |
| NS5b | 555 | Y555H |  |  |  |  |  |  |
| NS5b | 556 | S556G/R | S556G/R |  |  |  |  |  |
| NS5b | 557 | G557R |  |  |  |  |  |  |
| NS5b | 558 | G558R | G558R |  |  |  |  |  |
| NS5b | 559 | D559G/N | D559G/N |  |  |  |  |  |
| NS5b | 561 | Y561H/N |  |  |  |  |  |  |

**Supplementary Table 1**. Table of known resistant variants screened for in this study.

**Supplementary Table 2**. Samples classified as mixed infections and genotype switches. Sequence information including the average sequence depth for each genome and its corresponding genotype are shown for each longitudinal sample.

| name | sequence | location relative to H77 | direction |
| --- | --- | --- | --- |
| oligo dA20 | AAAAAAAAAAAAAAAAAAAA | 9418-9437 | R |
| RT primer | GGCGGAATTCCTGGTCATAGCCTCCGTGAA | 8616–8645 | R |
| PCR1-F | GGGTCGCGAAAGGCCTTGTGGTACTGCC | 266-293 | F |
| PCR1-R | CAGGAAACAGCTATGACGGCGGAATTCCTGGTCATAGCCTCCGTGAA | 8616–8645 | R |
| PCR2-F | GTACTGCCTGATAGGGTGCTTGCGAGTGCC | 286-315 | F |
| PCR2-R | AATTCCTGGTCATAGCCTCCGTGAAGACTC | 8611–8640 | R |

**Supplementary Table 3**. Primers used in this study. For the first of two PCR reactions in the nested PCR assay, primers PCR1-F and PCR1-R were used, whereas in the second PCR reaction, PCR2-F and PCR2-R were used.


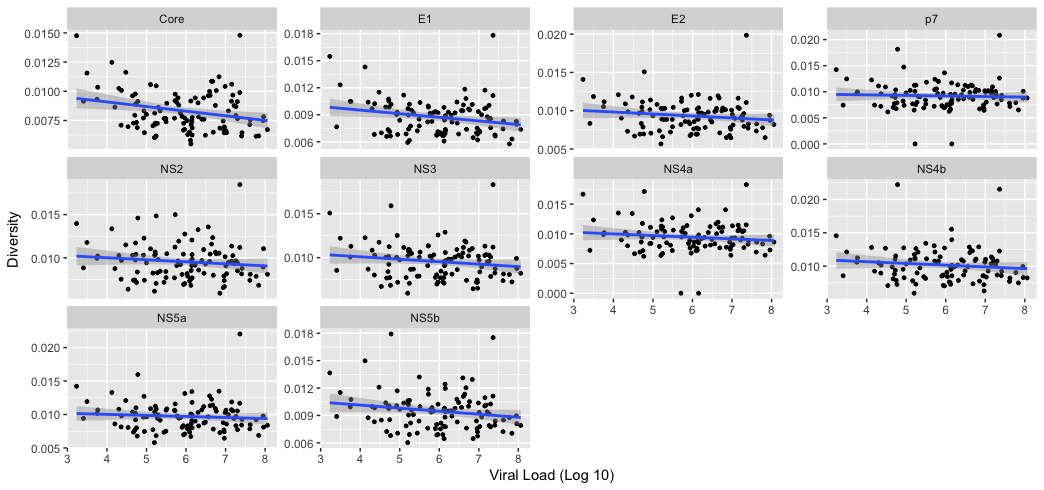


**Supplementary Figure 1**. Correlation between diversities and viral load (log_10_)

**Supplementary Figure 2**. **Correlation matrix of clinical variables**. Cells with ‘X’ indicate a p-value greater than 0.05. L6M represents last six months prior to sample collection.


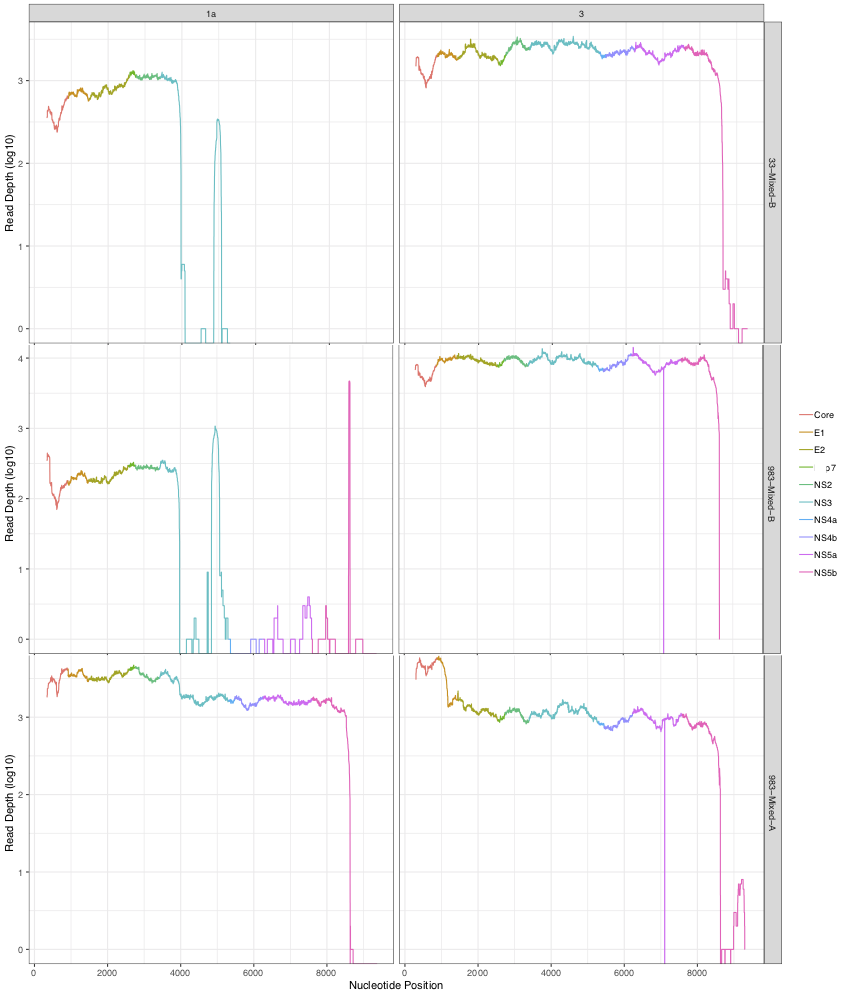


**Supplementary Figure 3**. **Depth of coverage for samples with mixed infections**. Figure columns represent each respective genotype and rows represent one sample from each individual.

**
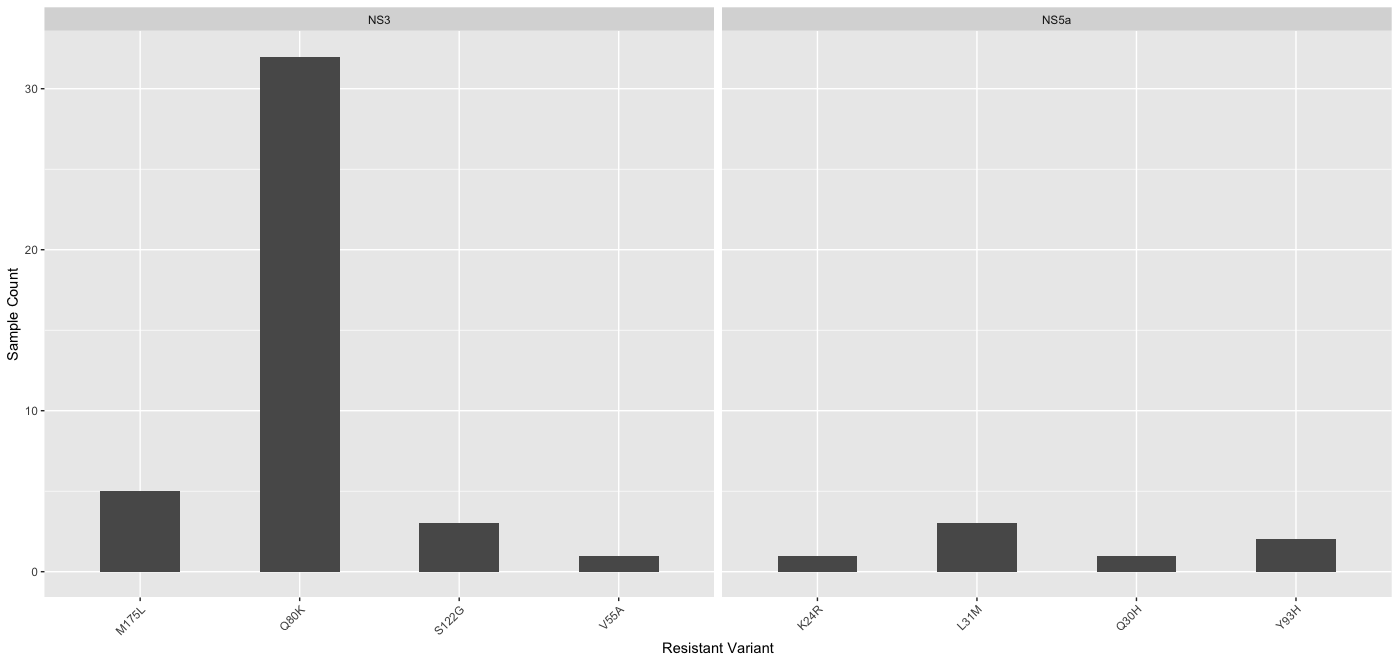
**

**Supplementary Figure 4. Resistance-associated site counts**. Counts of resistance-associated sites for the NS3 and NS5a genes for the samples in this study.


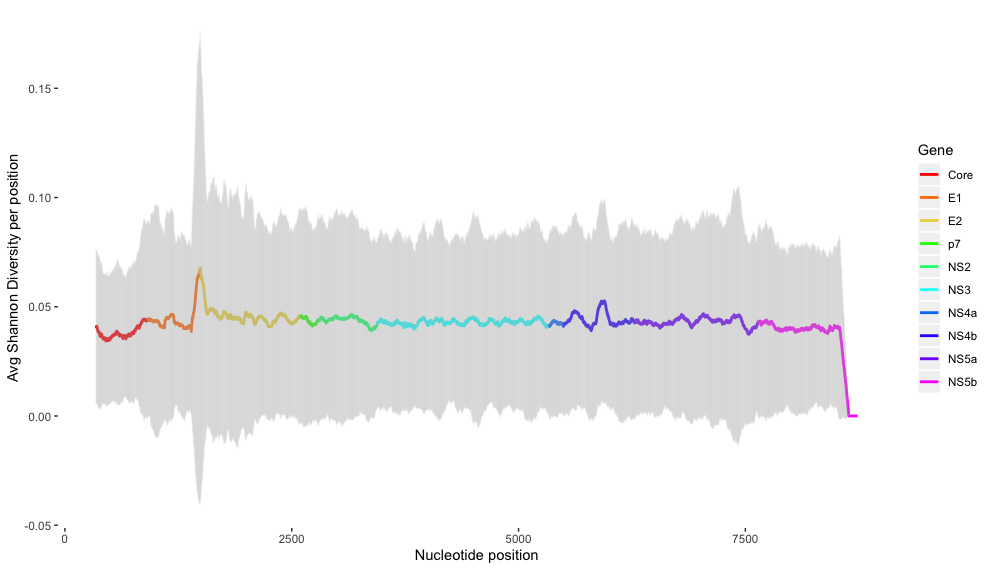


**Supplementary Figure 5. Shannon diversity across the HCV genome**. Median Shannon diversities per position for all samples in this study, normalized to 100 bp windows

**
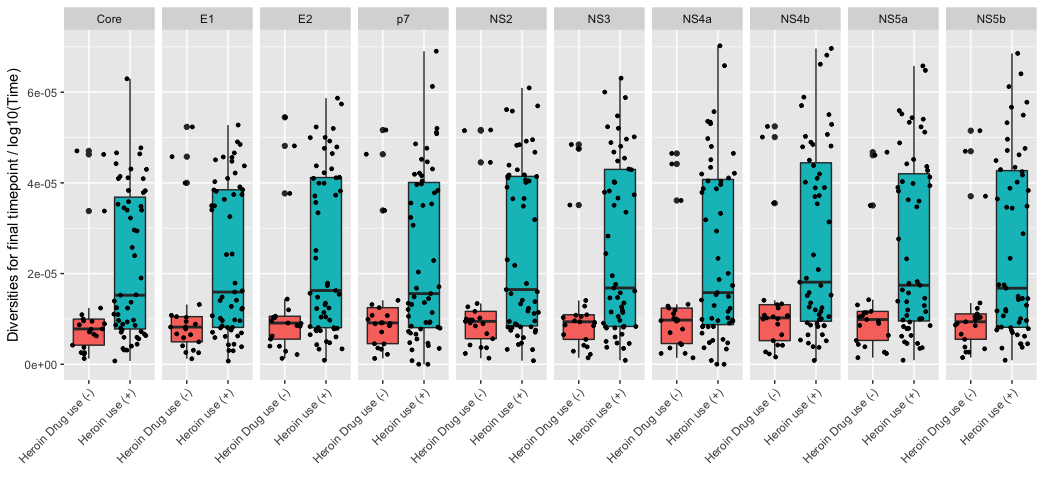
**

**Supplementary Figure 6** Comparing diversities for those who do and do not use heroin for the first time points of each individual. All genes except the NS2 and p7 genes displayed significantly elevated diversities relative to those not engaging in recent heroin use (as per a Mann-Whitney Wilcoxon test).


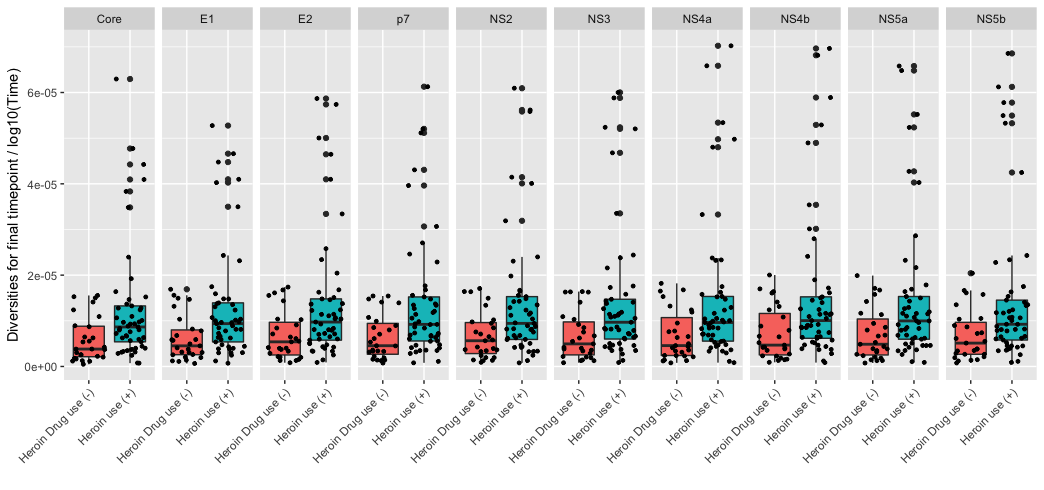


**Supplementary Figure 7** Comparing diversities for those who do and do not use heroin for the final time points of each individual. Only the Core, E1, and NS3 reached significantly elevated levels relative to those not engaging in recent heroin use (as per a Mann-Whitney Wilcoxon test).


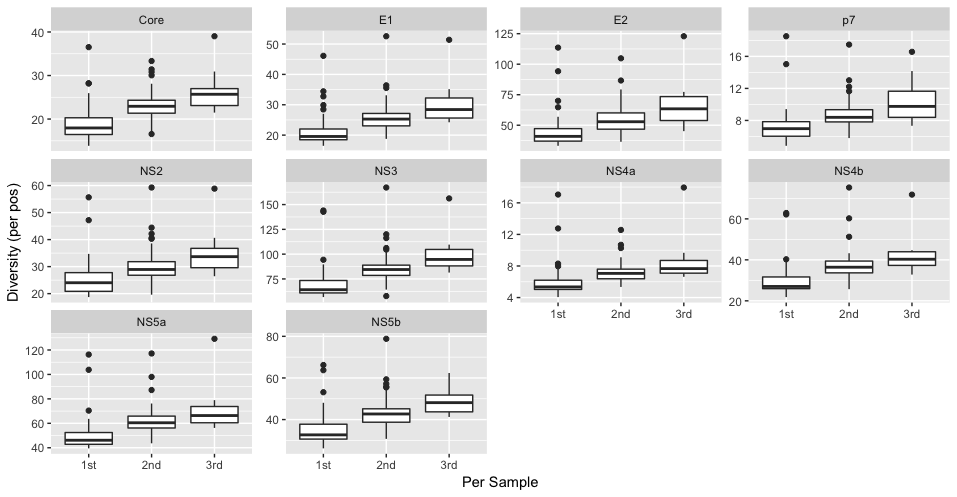


**Supplementary Figure 8. Shannon diversity across the time points**. Median Shannon diversity per time point (1st, 2nd, 3rd) separated by gene.

**
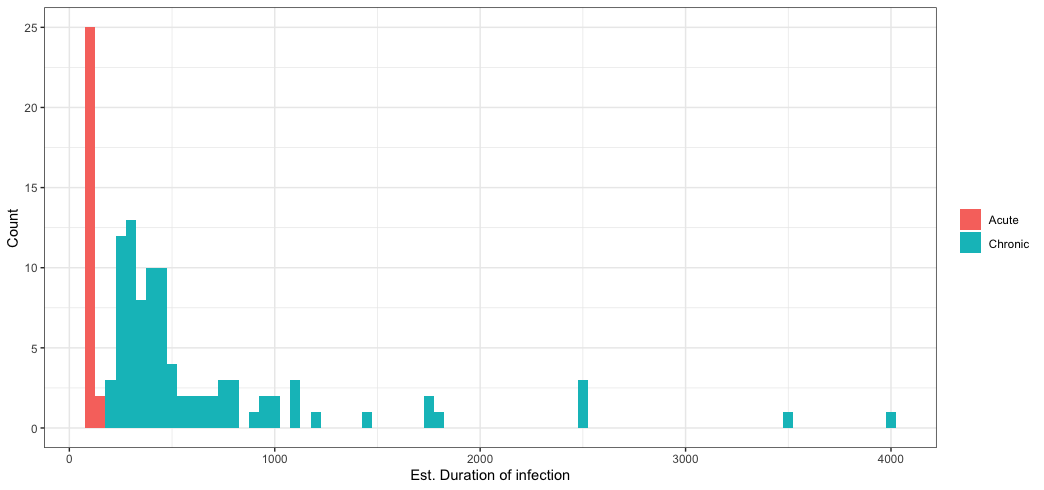
**

**Supplementary Figure 9.** Histogram of the estimated duration of infections for the samples included in this study. Note that the majority of chronic infections (greater than six months infected) are in close temporal proximity to acutely infected individuals (less than 6 months).


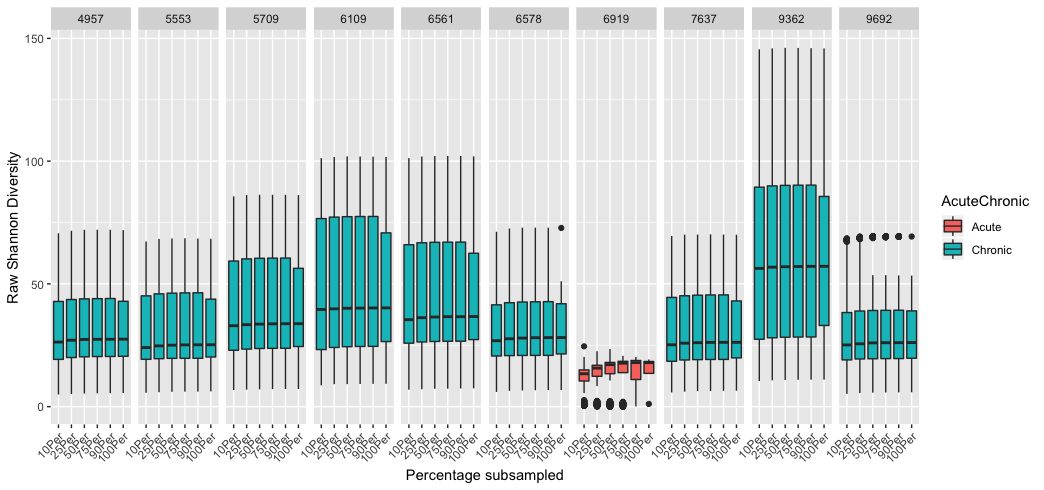


**Supplementary Figure 10.** Diversities for the random sub-sampling of 10 samples from this study with above average depths of coverage (> 8,000) relative to samples from this study. Reads were randomly sub-sampled at 10% (10Per), 25% (25Per), 50% (50Per), 75% (75Per), and 100% (100Per) 100 times (except for 100% which was performed once). The large range of diversities for each percentage is due to the fact that all genes are included in each bar.

**
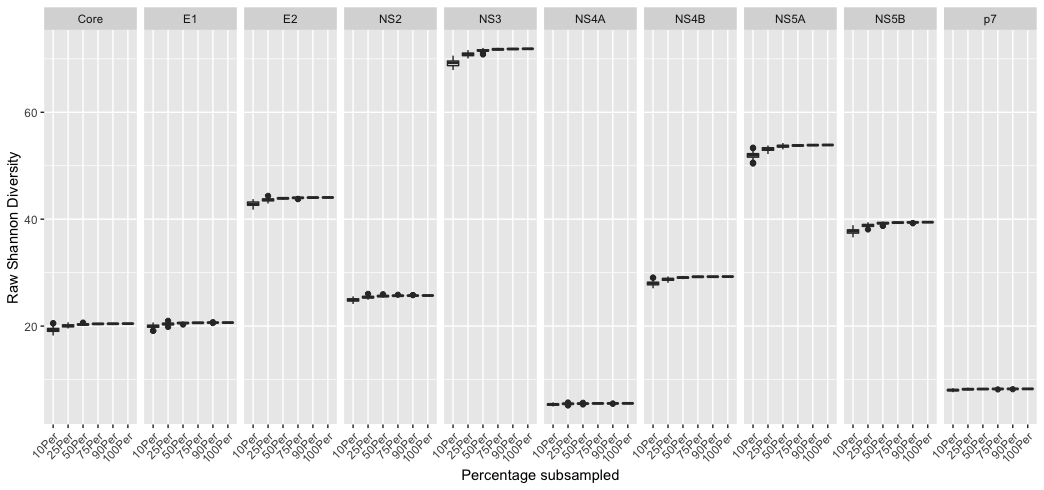
 Supplementary Figure 11.** Diversities for the random sub-sampling of one sample (4957), per gene . Reads were randomly sub-sampled at 10% (10Per), 25% (25Per), 50% (50Per), 75%(75Per), and 100% (100Per) 100 times (except for 100% which was performed once).


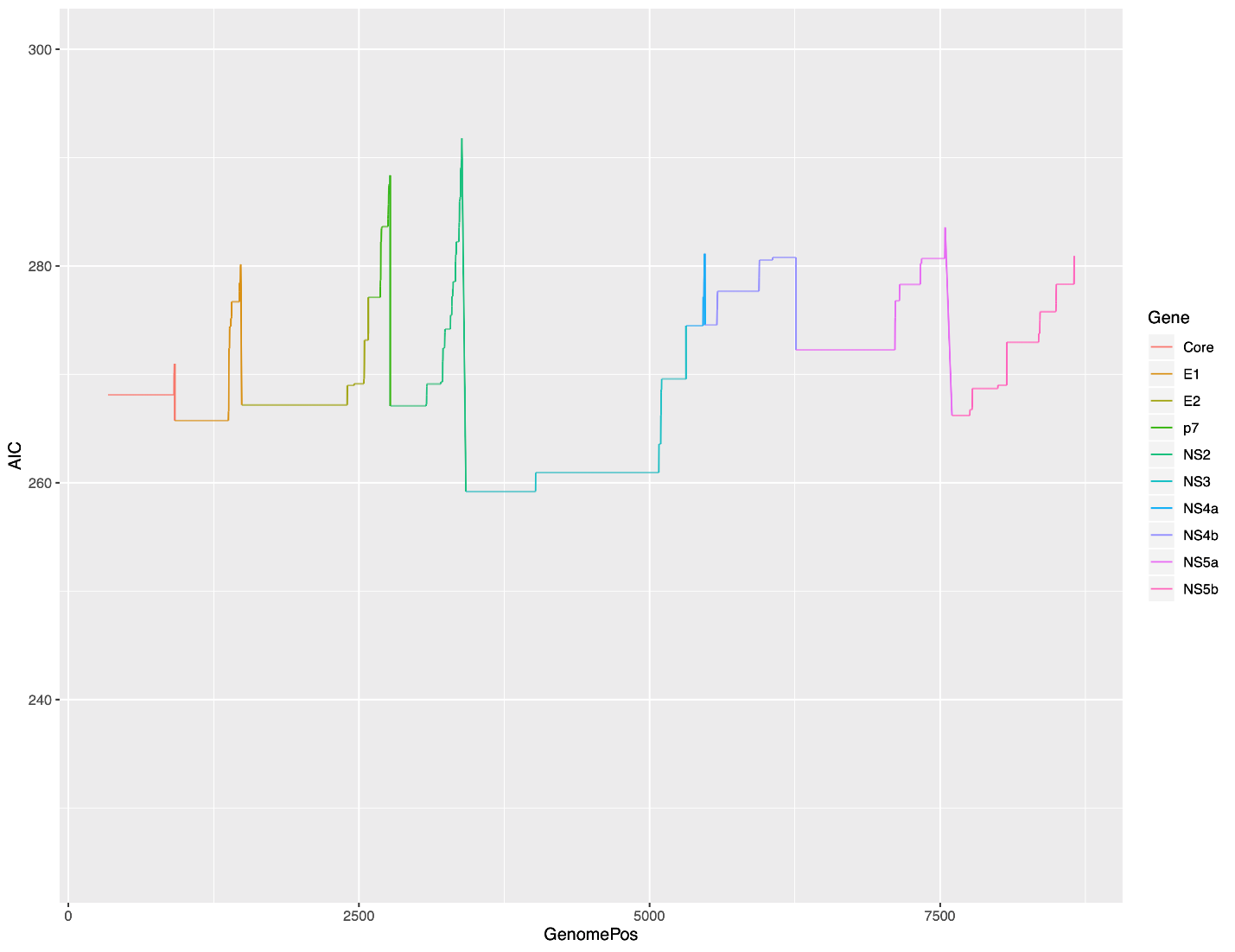


**Supplementary Figure 12.** The distribution of AIC values obtained from each linear model predicting duration of infection from viral diversity for each 100 bp window. For each overlapping window the minimum AIC was retained. The lowest AIC values were reached within the NS3 region.


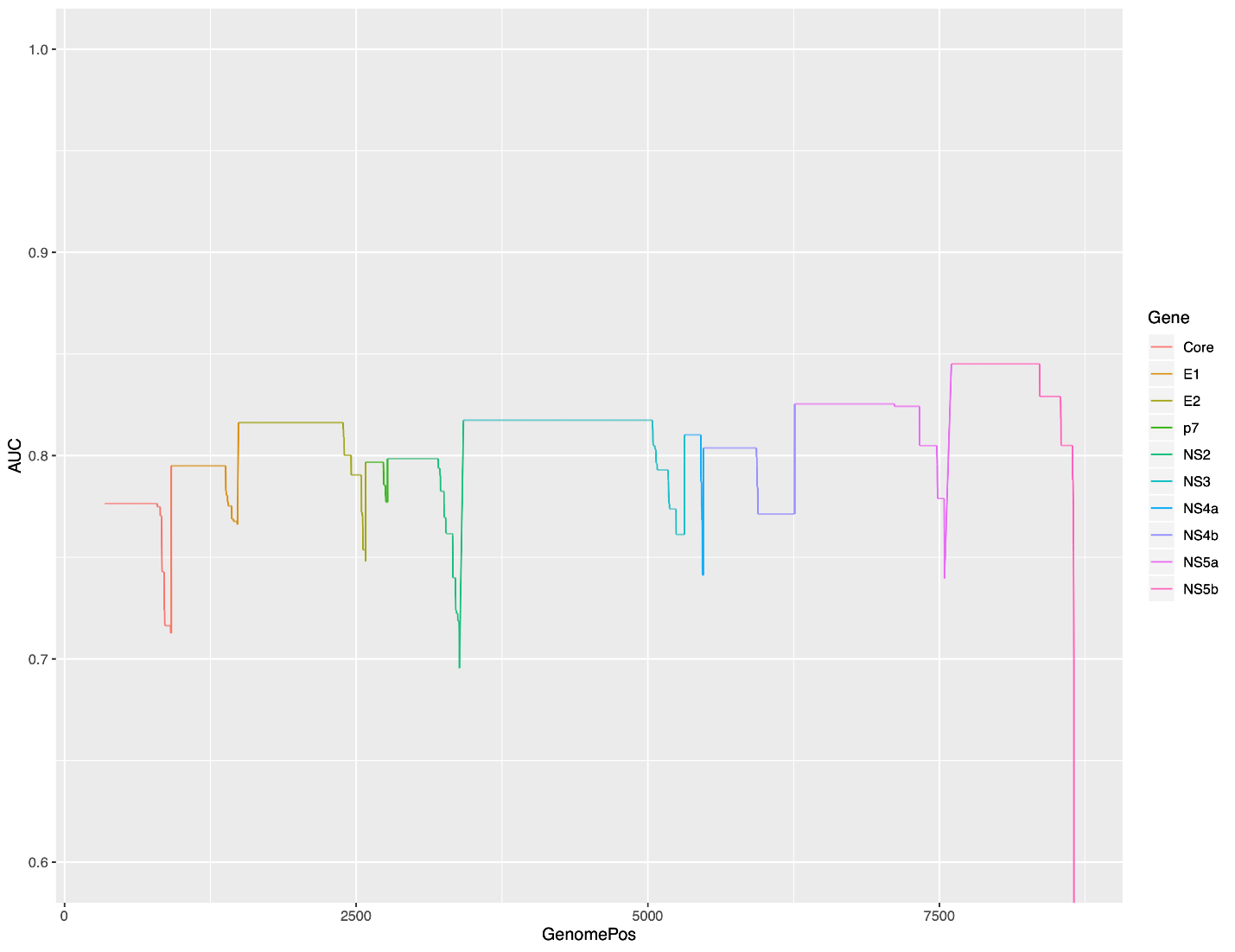


**Supplementary Figure 13.** The distribution of the area under the receiver operating characteristic curves (AUC). The highest AUC was reached in the NS5b region. For each overlapping window the maximum AUC was retained.

4

SE = - ∑ *p_i_ ln p_i_*

*i = 0*

**Equation 1**. Shannon entropy used for analysis, the proportion (p_i_) of each nucleotide at each position is multiplied by the natural logarithm of the same proportion
